# Adaptation and agreement analysis of an ADHD symptom observation instrument for adults with ASD in a specific residential setting

**DOI:** 10.1192/j.eurpsy.2025.665

**Published:** 2025-08-26

**Authors:** P. Ulsen, L. R. R. Carreiro, S. M. Blascovi-Assis, M. A. Munoz

**Affiliations:** 1 Universidad de Granada, Granada, Spain; 2 Universidade Presbiteriana Mackenzie, São Paulo, Brazil

## Abstract

**Introduction:**

Attention Deficit Hyperactivity Disorder (ADHD) is a neurodevelopmental disorder characterized by attention problems, hyperactivity, and impulsivity that can persist into adulthood. Autism Spectrum Disorder (ASD) is also a neurodevelopmental disorder with deficits in communication and social interaction, as well as restricted interests and stereotyped behaviors. High comorbidity between ASD and ADHD suggests an overlap of the two disorders, which can exacerbate the severity of both conditions.

**Objectives:**

Adapt and analyze the inter-rater reliability of a protocol for observing symptoms of ADHD and evaluate the presence of these symptoms in a sample of adults diagnosed with ASD.

**Methods:**

The study was conducted at a residential center dedicated to the care of adults with ASD in Granada - Spain. The experimental procedure was approved by the Ethics Committee of the University of Granada. The adapted version of the protocol for observing symptoms of ADHD in adults with ASD, consisting of 19 items that evaluate three areas: inattention; hyperactivity, and impulsivity. The response options were: Never=1, Rarely=2, Sometimes=3, Often=4, Very Often=5. The questionnaire was translated into Spanish by experts. Items were adapted to better reflect the daily activities in the residential setting. Behavior of the participants was recorded by 2 observers, the Caregiver and Technical Staff (Psychologists or Occupational Therapists), who had known the participant for at least 6 months. Each professional received training on how to fill it out. Analyses consisted of a reliability test to evaluate the agreement and consistency of the observers’ responses using the Intraclass Correlation Coefficient (ICC) through a two-factor Mixed Fixed-Effects Model. The presence of ADHD symptoms in the sample of 20 participants (14 men) diagnosed with ASD was tested descriptively.

**Results:**

The results for agreement on ICC were: 9 items were below .5 (poor); 3 items between .5 and .75 (moderate); 7 items between .75 and .9 (good). For consistency, the results were: 7 items below .5; 5 items between .5 and .75; 7 items between .75. The ICC result for absolute agreement (ICC = 0.943; 95% CI = [0.900, 0.973]; p < 0.001) was .94, indicating reliability above 0.9 (excellent). The ICC result for consistency (ICC = 0.949; 95% CI = [0.911, 0.976]; p < 0.001) was .94, with reliability above 0.9. The mean scores for the presence of ADHD symptoms indicate that both Caregivers and Technicians most frequently rated the items as 2 (Rarely) and 3 (Sometimes).

**Conclusions:**

In the overall instrument analysis, the results of interobserver agreement and response consistency reinforce that the adapted instrument is useful to identify ADHD behaviors in individuals with ASD. The need to continue developing and adapting instruments to identify comorbidities between profiles remains important.

**Disclosure of Interest:**

None Declared

